# Determining if Positive Predictive Value using Laboratory Risk Indicator for Necrotising Fasciitis is Applicable in Malaysian Patients with Necrotising Fasciitis

**DOI:** 10.5704/MOJ.1707.005

**Published:** 2017-07

**Authors:** A Syed, T Alvin, A Fazrina, R Abdul

**Affiliations:** Department of Orthopaedics, Hospital Tuanku Ja'afar Seremban, Seremban, Malaysia

**Keywords:** necrotising fasciitis, LRINEC, necrotising soft tissue infections, positive predictive value, sensitivity

## Abstract

**Introduction:** Necrotising fasciitis (NF) is a rapidly progressive infection of the subcutaneous tissue and fascia which spreads rapidly. The scoring system of Laboratory Risk Indicator for Necrotising Fasciitis (LRINEC) developed by Wong *et al* has been proposed as a tool for distinguishing NF and other soft tissue infections (STI) in Singapore. We set out to establish whether the LRINEC score is applicable in our Malaysian setting.

**Materials and Methods:** A cross sectional study of all patients admitted to our hospital diagnosed with NF or To Rule Out NF (TRO NF) between January 1st 2016 to 30th June 2016. The sensitivity, specificity, positive and negative predictive values were then calculated for LRINEC score of ≥ 6 and ≥ 8.

**Results:** Fourty-four patients were identified with the diagnosis of NF or TRO NF in the study. Twenty-seven patients (61.4%) were deemed post-operatively as having NF and 17 patients (38.6%) not having NF. A sensitivity of 59.3% and specificity of 47.1% when a LRINEC score of ≥ 6 was taken with positive predictive value (PPV) of 64.0% and the negative predictive value (NPV) of 42.1%. When score ≥ 8 was taken, the sensitivity was 48.1% and specificity of 58.8% with PPV of 65% and NPV of 41.7%.

**Conclusion:** The low sensitivity and low PPV achieved in this study as well as other studies makes the LRINEC score unsuitable to be used solely to distinguish NF with other soft tissue infections.

## Introduction

Necrotising fasciitis (NF) is a rapidly progressive infection of the subcutaneous tissue and fascia which spreads along the fascial plane. Often it is accompanied by severe systemic toxicity, seen as haemorrhagic bullae as part of toxicepidermal necrolysis, septic shock and progressive multi organ failure^1^. It is a potentially lethal disease with a high mortality rate ranging from 9%-29%^2–5^ as reported in the literature.

Early recognition, aggressive debridement of all necrotic fascia and subcutaneous tissues as well as early commencement of intravenous antibiotics are major prognostic determinants and delay in operative debridement has shown to increase mortality rate^2,6,7^.

The diagnosis of NF if mainly established from a high index of suspicion upon clinical presentation. It is however, difficult to clinically distinguish it from cellulitis or abscesses early in its evolution. Modalities such as Magnetic Resonance Imaging (MRI) and frozen section biopsy have been shown to be useful in early recognition of the disease^8,9^. However, due to limitations of cost and availability, their applicability has been limited.

The scoring system Laboratory Risk Indicator for Necrotising Fasciitis (LRINEC) by Wong *et al*^10^ was proposed as a tool for distinguishing NF and other soft tissue infections (STI) in Singapore. It is the purpose of this study to establish whether the LRINEC score is applicable to the local setting.

## Materials and Methods

We conducted a cross-sectional study involving a retrospective analysis of all patients admitted to Hospital Tuanku Ja’afar, Seremban, diagnosed with NF or To Rule Out NF (TRO NF) between January 1st 2016 to 30 June 2016. Data were extracted from admission logs and from perusal of patients’ case notes. Blood tests taken on admission were used to calculate the LRINEC score (Table I) as per Wong *et al* original study. A 2x2 table was then tabulated to determine the sensitivity, specificity, positive and negative predictive values as well as charting of the receiver operating characteristics (ROC) curve. Patients with inadequate investigations taken to complete the LRINEC score were excluded from the study. The diagnosis of NF was established intraoperatively by the operating surgeon. A LRINEC score of ≥6 was considered to indicate moderate risk of NF and a score of ≥8 was considered to have high risk of NF in accordance with the study by Wong *et al.*

**Table I:** Laboratory Risk Indicator for Necrotising fasciitis (LRINEC) score

**Variable (Units)**	**Score**
CRP (mg/L)	
<150	0
≥150	4
TWC (mm3)	
<15	0
15-25	1
>25	2
Haemoglobin (g/Dl)	
>13.5	0
11-13.5	1
<11	2
Sodium (mmol/L)	
≥135	0
<135	2
Creatinine (μmol/L)	
≤141	0
>141	2
Glucose (mmol/L)	
≤10	0
>10	1

## Results

**Fig. 1:**
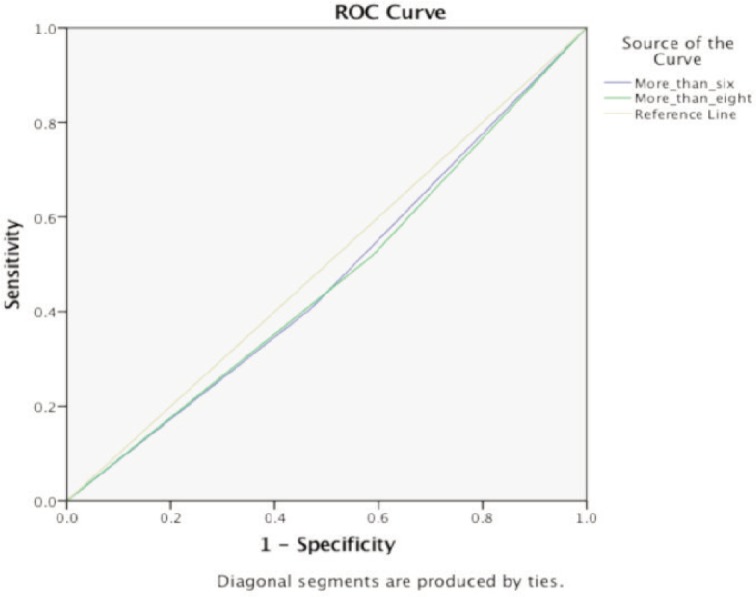
Receiver Operating Curve for score ≥ 6 and ≥ 8.

Fourty-four patients were identified with the diagnosis of NF or TRO NF. 29 of the patients were male (65.9%) and 15 females (34.1%). There were 20 Malays and Indians (45.5%), and two Chinese patients (4.5%) and two other races (4.5%) (Table II). The mean age was 54.9. Twenty-five patients had a LRINEC score of ≥6 (56.8%) and 19 patients had a LRINEC score of <6 (43.2%). Twenty-seven patients (61.4%) were deemed post operatively as having NF and 17 patients (38.6%) not having NF. Cross tabulation of the data obtained revealed a sensitivity of 59.3 % and specificity of 47.1% when a LRINEC score of ≥ 6 was taken (Table III) (95% CI of 42.2% -70.3%). The positive predictive value (PPV) obtained was 64.0% and the negative predictive value (NPV) was 42.1%. When a score ≥ 8 is taken the sensitivity drops to 48.1% and specificity increases to 58.8% (95% CI 31.7% - 59.9%) while the PPV is at 65% and NPV at 41.7%. (Table IV). A receiver operating characteristic (ROC) curve (Fig. 1) was charted for LRINEC score ≥ 6 and ≥ 8 with area under the curve analysis of 0.468 and 0.465 respectively (Table V). Nine amputations were carried out amounting to 20.9% of the sample while four patients died during the course of the study carrying a mortality rate of 20.4%.

**Table II:** Demography of patients

**Variables**	**Percentage (%)**
Gender	
Male	65.9
Female	34.1
Total	100
Race	
Malay	45.5
Chinese	4.5
Indian	45.5
Others	4.5
Total	100

**Table III:** Sensitivity and specificity when LRINEC score more than 6

	**NF**	**Not NF**	**Total**	
LRINEC Score				
≥6	16	9	25	Sensitivity 59.3 %
<6	11	8	19	Specificity 47.1 %
Total	27	17	44	

**Table IV:** Sensitivity and specificity when LRINEC score more than 8

	**NF**	**Not NF**	**Total**	
LRINEC Score				
≥8	13	7	20	Sensitivity 48.1 %
<8	14	10	24	Specificity 58.8 %
Total	27	17	44	

**Table V:** Area under the curve(AUC) for ROC curve

**LRINEC score**	**AUC**	**95% CI**
≥ 6	0.468	0.29-0.64
≥ 8	0.465	0.28-0.64

## Discussion

The LRINEC score was first proposed in 2004 by Wong *et al* with the purpose of distinguishing NF from other soft tissue infections using routine biochemical tests. Based on its study, a cutoff value of 6 showed a PPV of 92% and NPV of 96%^10^. However, we gathered a sensitivity of 59.3% and specificity of 47.1% with PPV of 64% and NPV of 42.1% when a LRINEC score of ≥ 6 is taken. These are similar to the results achieved by Al-Hindawi *et al*^11^ and Liao *et al*^12^with the sensitivity reported as 43.2% and 59.2% respectively. Due to the life threatening nature of the disease, the LRINEC scoring system is too insensitive to be relied on for diagnosing NF.

The infection carries a mortality rate of 14-40% in some case series^13,14^. Therefore having a low sensitivity exposes patients to delay in diagnosis and treatment. Furthermore, a Receiver Operating Curve (ROC) graph charted, shows an area under the curve less than 0.5, making the LRINEC scoring not reliable to diagnose NF early in its evolution. The high levels of the biochemical tests would also be present in other conditions, like abscesses, catheter related sepsis, and pneumonia and do not specifically represent NF. It was also noted that during this study that the patients usually had multiple preexisting co-morbidities as well as other possible foci of infection that may cause higher LRINEC scores.

However, the authors of this study acknowledge that this retrospective study is limited by its small size as well as the relatively short duration of study. These results are also only reflective of the local demographics of the small geographic area of the city of Seremban in Negeri Sembilan, Malaysia, and is not a multicentre study. Despite that, the LRINEC score is probably too insensitive and not robust enough to be used alone in diagnosing NF. It is also surprising to note that 52.9% of patients with LRINEC score of ≥ 6 were postoperatively diagnosed as not having NF making the LRINEC score unreliable to distinguish NF from other soft tissue infections.

## Conclusion

Necrotising fasciitis is an aggressive disease that requires urgent therapeutic intervention to improve patient’s outcome. The alarmingly high rate of false negatives, its low sensitivity and low PPV makes the LRINEC score unreliable to be used solely to distinguish NF with other soft tissue infections in the local setting.
